# *Cauliflower mosaic virus* transactivator protein (TAV) can suppress nonsense-mediated decay by targeting VARICOSE, a scaffold protein of the decapping complex

**DOI:** 10.1038/s41598-019-43414-0

**Published:** 2019-05-07

**Authors:** Nina Lukhovitskaya, Lyubov A. Ryabova

**Affiliations:** 1 Institut de biologie moléculaire des plantes, CNRS, Université de Strasbourg, Strasbourg, France; 2 Present Address: Division of Virology, Department of Pathology, University of Cambridge, Cambridge, CB2 1QP UK

**Keywords:** Plant immunity, Plant signalling

## Abstract

During pathogenesis, viruses hijack the host cellular machinery to access molecules and sub-cellular structures needed for infection. We have evidence that the multifunctional viral translation transactivator/viroplasmin (TAV) protein from *Cauliflower mosaic virus* (CaMV) can function as a suppressor of nonsense-mediated mRNA decay (NMD). TAV interacts specifically with a scaffold protein of the decapping complex VARICOSE (VCS) in the yeast two-hybrid system, and co-localizes with components of the decapping complex in planta. Notably, plants transgenic for TAV accumulate endogenous NMD-elicited mRNAs, while decay of AU-rich instability element (ARE)-signal containing mRNAs are not affected. Using an agroinfiltration-based transient assay we confirmed that TAV specifically stabilizes mRNA containing a premature termination codon (PTC) in a VCS-dependent manner. We have identified a TAV motif consisting of 12 of the 520 amino acids in the full-length sequence that is critical for both VCS binding and the NMD suppression effect. Our data suggest that TAV can intercept NMD by targeting the decapping machinery through the scaffold protein VARICOSE, indicating that 5′-3′ mRNA decapping is a late step in NMD-related mRNA degradation in plants.

## Introduction

mRNA decay controls endogenous RNA degradation in response to environmental, developmental and physiological signals^1^. Such mRNA quantity and quality control represents an additional step of co-translational regulation that limits mRNA abundance by degrading faulty cellular mRNAs, thus preventing the production of aberrant proteins. These strategies of mRNA degradation are based on the coordinated action of specific endo- and exo-nucleolytic complexes that recognize transcripts as substrates due to the presence of cis-acting instability elements such as AU-rich elements (ARE), nonsense-mediated decay (NMD) signals or *trans*-acting mRNA destabilizing factors. mRNA degradation is initiated mainly by shortening of the 3′ poly(A) tail (deadenylation), which leads to the removal of the 5′-cap structure (decapping) and subsequent proteolytic degradation by a 5′ to 3′ exonuclease^2^. Alternatively, deadenylated RNA can be digested from 3′ to 5′ by the exosome^3^.

In posttranscriptional control, up to 20% of endogenous transcripts, including functional protein-coding as well as noncoding RNAs, are NMD targets due to the presence of NMD-eliciting signatures such as a long 3′UTR (more than 300 nt), a long upstream open reading frame (uORF >30 codons) within the 5′UTR, and a premature termination codon (PTC) positioned >50–55 nt upstream of the last exon-exon junction in the spliced mRNA^4–7^. Increasing data suggest a general role for NMD in the identification of PTC-containing mRNAs and their degradation^8^. In mammals and plants, the conserved core of the NMD machinery consists of UP-FRAME SHIFT 1 (UPF1), UPF2, UPF3, and SUPRESSOR WITH MORPHOLOGICAL EFFECT ON GENITALIAs (SMGs)^8,9^. However, the late steps of NMD-elicited mRNA degradation are not well defined and are thought to proceed either via the SMG7-UPF1 or the UPF1-XRN4 pathway^9^. Likewise, AtXRN4, the cytoplasmic ortholog of mammalian XRN1, seems not to be essential for plant NMD^10,11^ raising questions relevant to the mechanism by which NMD targets are degraded to nucleotides. In Arabidopsis, the decapping activity is performed by the core of the decapping complex composed of DCP1, DCP2, and VARICOSE (VCS)^12^. The decapping complex components are required for decapping activity, processing body (PB) formation and translational repression^13^. It was recently shown that VCS contributes to decay of more than 50% of the transcriptome, especially to decay of short-lived mRNAs^14^. Deadenylation requires the conserved carbon catabolite repressor 4 (CCR4) complex^15^.

RNA silencing functions as major host defence mechanism against viruses in plants, fungi and invertebrates^16–18^. NMD contributes to plant innate immunity by controlling expression of defence-related genes^7,19^ and stability of RNAs with long 3′UTRs in positive-strand RNA viruses of the *Alphaflexiviridae* and *Tombusviridae*^20^. In mammals, NMD restricts replication of some positive-strand RNA viruses such as *Semliki Forest Virus* (SFV)^21^. Viruses have developed multiple strategies to protect their transcripts from the host surveillance machinery. *Tobacco mosaic virus* (TMV) activates RNA decay pathways to down-regulate RNA silencing and modulate symptom development^22^. Hepatitis C virus (HCV) encodes NMD suppressor proteins that affect the integrity of the EJC complex^23^. The most frequent counter-decay strategies of viruses are based on the use of RNA structural features, i.e., cis-acting RNA elements that can prevent viral RNA recognition by the cellular surveillance machinery and thus avoid RNA degradation by decay-related enzyme complexes^24–28^. For example, *Rous sarcoma virus* (RSV) possesses a specific cis-RNA element termed the RNA stability element (RSE) that recruits the polypyrimidine tract binding protein 1 (PTBP1), preventing UPF1 binding to RSE^29^, while proteins from human T-lymphotropic virus type 1 (HTLV-1) down-regulate NMD via binding to its core components^30,31^. Taken together, those data suggest that NMD might be considered as an evolutionarily conserved antiviral mechanism^28^.

*Cauliflower mosaic virus* (CaMV)—a member of the genus Caulimovirus—infects crucifer species including *Arabidopsis* and some solanaceous species^32^. CaMV contains a circular, double-stranded DNA genome that replicates via reverse transcription of the 35S pregenomic RNA^32^ (pgRNA). The polycistronic 35S pgRNA is translated under control of P6/translational activator/viroplasmin (TAV) to produce the six viral proteins (P1–P6), while TAV is produced mainly from the monocistronic 19S RNA^33,34^. TAV counteracts plant defence mechanisms^35–37^, suppresses RNA silencing^35,38,39^ and is likely to be involved in the assembly and transport of CaMV particles^40–43^. The 35S pgRNA undergoes alternative splicing^44,45^. In total, spliced 35S pgRNAs represent approximately 70% of total viral RNA in CaMV-infected plants, and about 30% of the pgRNA is exported to the cytoplasm in its unspliced form^44,45^. Thus, splicing events within the leader region and ORFs I and II generate multiple unspliced and differentially spliced polycistronic mRNAs^44,45^ that are potential targets for cellular RNA surveillance machinery. How the 35S pgRNA and its spliced versions comprising consecutive open reading frames and exon-exon junctions escape the mRNA surveillance system of the host cell remains an open question.

The six long ORFs of the polycistronic 35S mRNA are translated via a reinitiation mechanism, which is uncommon in eukaryotes. To accomplish reinitiation after long ORF translation, TAV recruits the components of the host translation machinery, including reinitiation supporting protein (RISP) and eukaryotic translation initiation factor 3 (eIF3), to ribosomes translating viral RNA, and activates a target of rapamycin (TOR) to maintain the high phosphorylation status of both proteins on the polysomes^46–49^. Although the role of the key growth-regulating TOR kinase in plant translation initiation is not yet known, TOR, when activated by TAV or plant hormone auxin, promotes translation reinitiation^50,51^. The presence of multiple uORFs that differ in size, length, and nature within the mRNA 5′UTR can significantly decrease the efficiency of reinitiation at the main ORF and thus trigger mRNA degradation. Thus TOR can overcome uORF repression pressure by up-regulating uORF-mRNA translation.

Here, we address the mechanism (s) whereby CaMV suppresses mRNA decay in plants. We demonstrate that TAV can behave as a pathogen effector due to its interaction with a scaffold protein of the decapping complex, VARICOSE, to antagonize NMD-targeted degradation of cellular mRNAs.

## Results

### Translation transactivator/viroplasmin (TAV) specifically binds VARICOSE (VCS) via its C-terminal α-helix

**Figure 1:**
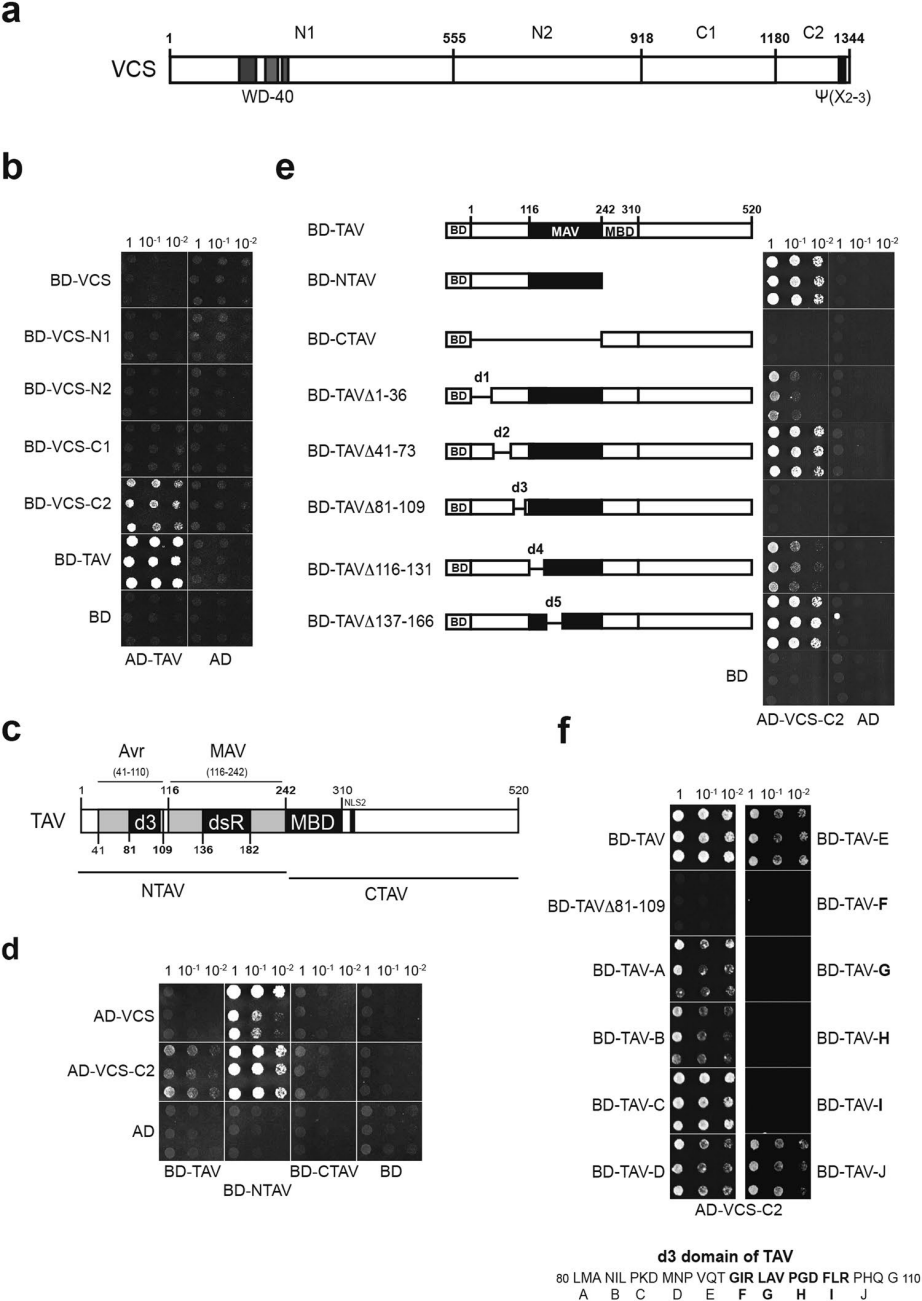
Evidence for interactions between translation transactivator (TAV) and VARICOSE protein. (**a**) Schematic representation of Arabidopsis VCS protein and its N1, N2, C1 and C2 domains. The amino acid numbers at the cut-off borders are shown. (**b**) Yeast two-hybrid (Y2H) interactions between TAV fused to Gal4 activation domain (AD) and VCS N1, 2 and C1, 2 domains fused to Gal4 binding domain (BD). Equal OD600 units and 1/10 and 1/100 dilutions were spotted from left to right and incubated for 2 days. (**c**) Schematic representation of TAV, its C- and N-terminal domains. TAV conserved domains, avirulence (Avr), minimal transactivation (MAV) domain and multiple protein binding (MBD), including d3 and double-stranded RNA binding (dsR) motifs, are shown. (**d**) Y2H interactions between AD-VCS, AD-VCS-C2 domains and TAV and its N- and C-terminal domains. (**e**) Mapping of TAV interaction motif within its N-terminus. N-TAV deletion mutants d1-d5 are presented. (**f**) A 12-amino-acid region within the d3 motif is responsible for C-terminal VCS domain C2 binding. Mutants carrying three-amino-acid substitution mutations to alanines within the d3 motif fused to BD designated A–J are used for AD-VCS-C2 interaction.

Studies of TAV-activated polycistronic translation led to the observation that transient expression of TAV CM1841 (TAV) in plant protoplasts correlated with increased levels of long bicistronic mRNAs in contrast to monocistronic reporter mRNAs, indicating that TAV may interfere with specific cellular RNA surveillance pathways^34^. To investigate how TAV might affect specific mRNA accumulation, we examined a TAV partner, a scaffold of the decapping complex VARICOSE, identified by an *A*. *thaliana* cDNA library Y2H screen. Here, the C-terminal 1600 nt fragment of mRNA encoding a varicose-related (VCR) protein^12^ was selected with an amino terminal portion of TAV (NTAV, residues 26–242). *Arabidopsis* VCR is related to varicose (VCS) protein and both share 40% amino acid identity with the human ortholog Hedls/Ge-1^12^. Since a vcr null mutant is indistinguishable from wild type, suggesting that VCR may not be functional^52^, we chose VCS for further analysis. VCS and VCR retain some of the features of the Hedls/Ge-1 protein structure, such as the WD40 repeats within their N-terminal domains and the ψ(X2-3) repeats within their C-terminus, which is constructed of α-helical hairpin repeats arranged in helix–turn–helix hairpins^53^ (see Fig. 1a for schematic of VCS and Supplementary Fig. S1 for alignment of VCS, VCR and Hedls/Ge-1). TAV (see Fig. 1c for schematic of TAV) and VCS truncation and deletion mutants fused to the Gal4 binding (BD) or activation (AD) domains, respectively, were tested in the Y2H system to delineate regions important for binding. In order to identify the domains contributing to the interactions with TAV, we cloned four putative domains based on their predicted secondary structure: VCS-N1 (aa 1–555; the domain contains WD40 repeats), VCS-N2 (aa 556–917; the domain contains an S-rich linker), VCS-C1 (aa 918–1180) and VCS-C2 (1181–1341) (Fig. 1b). The cut-off borders of the deletions were preferentially at proline-glycine dipeptides, because proline is found only rarely within α-helices and therefore such deletions are expected to have a minimal effect on protein conformation. According to the Y2H system, entire TAV could bind to the VCS-C2 (BD-VCS-C2) region that adopts alpha-helical fold related to HEAT-repeat proteins (Fig. S1), while we fail to reveal Y2H interaction between entire proteins possibly due to C2 domain inaccessibility within the large VCS protein, or VCS/DCP1/DCP2 complex formation^12^. TAV, known to contain several domains—N-terminal domain (aa 1–116), a critical minimal transactivation domain (MAV) and the domain containing the zinc finger (aa 446–462)—are involved in self-assembly and formation of viral inclusion bodies in the cytoplasm of infected cells^54^, displayed strong dimerization signal as expected (Fig. 1a). Here, the TAV N-terminal, but not C-terminal, domain is responsible for VCS-C2 binding (Fig. 1d). The VCS C-terminal domain is critical for decapping complex activity; this domain was shown to be involved in VCS dimerization and binding to DCP2^12^.

However, N-TAV interacts with both entire VCS and VCS-C2 in Y2H (Fig. 1d). Taking advantage of published results on TAV deletion mutagenesis, we used TAV mutants carrying deletions within its N-terminus (d1–d5; Fig. 1e)^55^ to further delineate the regions of N-TAV involved in binding to the C-terminus of VCS in Y2H assays. An internal deletion of TAV (d3, aa 81–109) abolished VCS-C2 binding (Fig. 1e) suggesting that d3 is a critical motif for VCS binding. Although deletion of d1 or d4 led to a significant loss in TAV binding, interactions were not abolished. Thus, we limited the VCS binding site to the 30 amino acid d3 motif (aa 81–110) located immediately upstream of MAV. Interestingly, the d3 motif contributes to the TAV/P6 Avr domain (amino acids 41–110) implicated in CaMV pathogenicity and host range^56–59^.

**Figure 2:**
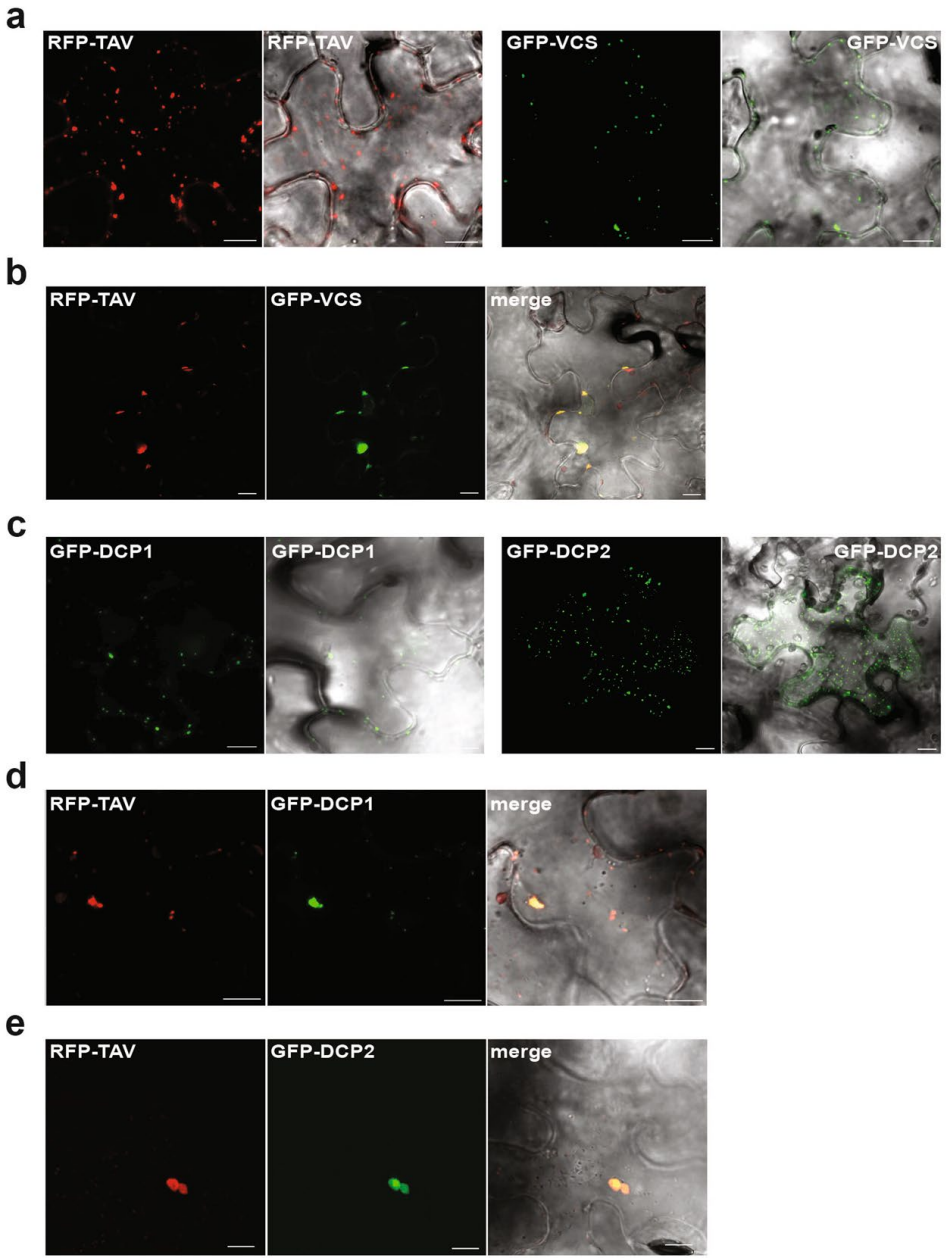
TAV viroplasms colocalize with components of the decapping complex in epidermal cells. (**a**–**e**) Subcellular localization of (**a**) RFP-TAV or GFP-VCS alone; (**b**) both RFP-TAV and GFP-VCS; (**c**) GFP-DCP1or GFP-DCP2 alone; (**d**) both RFP-TAV and GFP-DCP1; (**e**) both RFP-TAV and GFP-DCP2 transiently expressed in *N*. *benthamiana* epidermal cells. Merged images superimposed with optical bright field image are shown on the right. Scale bar, 10 µm.

Next, we further delineated the TAV d3 motif to assess specificity of TAV binding to the VCS C-terminal domain, and constructed a series of three amino acid alanine substitutions (Fig. 1f). Strikingly, only the substitution of 12 amino acids within the central region of the d3 motif abolished TAV binding to VCS C2, indicating the existence of a narrow TAV motif required for interaction with VCS. Thus, TAV binding to VCS involves the final C-terminal α-helix of VCS and a 12 amino acid motif within the d3 region of TAV. Expression levels of TAV, VCS and their modified or truncated proteins were controlled by immunoblot and no significant variations were observed (data not shown). We next conducted a co-localization assay to reveal whether TAV may co-localize with the decapping complex in the cytoplasm of *N*. *benthamiana* epidermal cells (Fig. 2). The core components of the decapping complex - VCS, DCP1 and DCP2 - are known to localize to small punctate cytoplasmic foci that are juxtaposed with putative processing bodies, P-bodies (PBs), which are the sites of accumulation of non-translated mRNAs^12^. Similar punctate cytoplasmic foci were marked with green fluorescent protein (GFP) tagged VCS (GFP-VCS; Fig. 2a), or GFP-DCP1, or GFP-DCP2 transiently overexpressed in the cytoplasm of *N*. *benthamiana* epidermal cells (Fig. 2c). In contrast, red fluorescent protein tagged TAV (RFP-TAV) forms cytoplasmic aggregates of different sizes and shapes^60^ (Fig. 2a), consistent with its role as a major component of viral inclusion bodies^54^. Upon transient overexpression of both RFP-TAV and GFP-VCS in *N*. *benthamiana* (Fig. 2b), GFP-VCS appeared as structures that overlap RFP-TAV aggregates in *N*. *benthamiana* epidermal cells, suggesting TAV association with P-bodies. Although we did not observe interactions between TAV and either DCP1 or DCP2 using the Y2H (data not shown), GFP-tagged DCP1 and DCP2 co-localized with RFP-TAV aggregates as well as GFP-VCS (Fig. 2d,e). We conclude that TAV may cause a redistribution of P-body components due to its binding to the decapping complex located within storage areas such as P-bodies (Fig. 2d).

### TAV overexpression in *Arabidopsis* is indispensable for NMD target mRNAs

**Figure 3:**
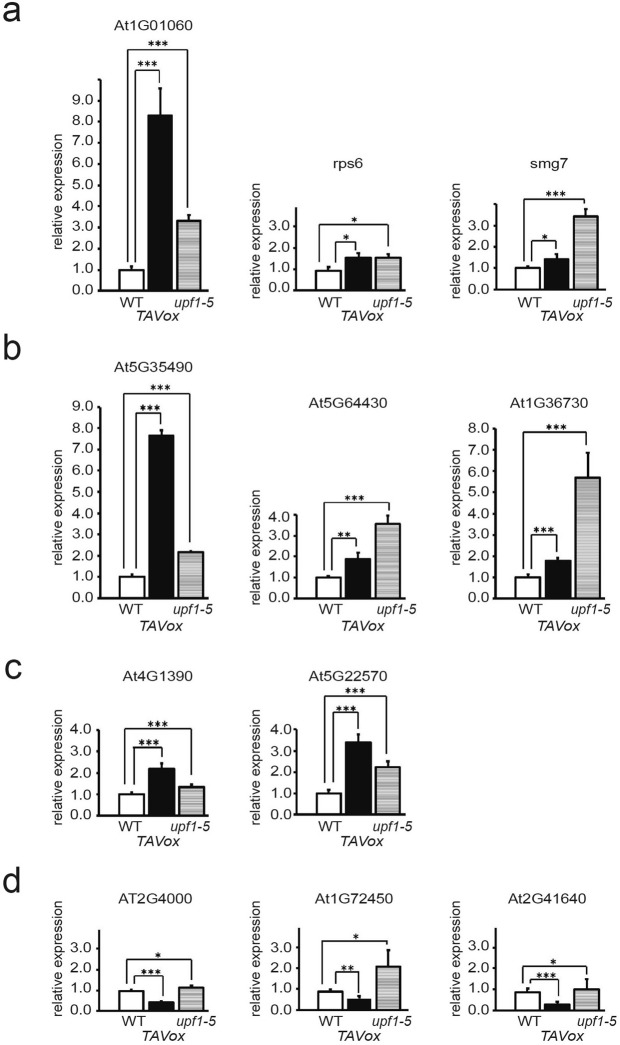
Attenuation of nonsense-mediated mRNA decay in TAV transgenic plants. (**a**–**d**) Quantitative qRT-PCR (qRT-PCR) analysis of known NMD target transcripts containing (**a**) premature termination codons (PTC; At1G01060^20^ and rps6^7^) or long 3′UTR and PTC (smg7^62^), (**b**) upstream ORFs (uORFs; At5G35490, At5G64430 and AtG36730^19^), or (**c**) unknown NMD triggering signals (AtG1390 and AtG22570^19^) and (**d**) ARE target transcripts containing AU-rich instability elements (At2g4000, At1g72450 and At2g41640)^63^ in WT, CM6 TAV transgenic line (*TAVox*) and *upf1-5* mutant Arabidopsis plants, normalized to *EXPLA1* (AT3G45970) and *SAND* (At2G28390). Values are expressed in arbitrary units and represent the mean +/− SD (n = 5), asterisks indicate a significant difference (*P < 0.05; **P < 0.01; ***P < 0.001, Student’s t test).

To estimate the functional significance of the interaction between TAV and VCS in the turnover of endogenous mRNAs, we assayed whether NMD-elicited or AU-rich instability element (ARE)-containing mRNA levels are altered in TAV-transgenic plants. We compared the steady state levels of selected endogenous mRNAs in three genotypes: WT Col-0, *upf1-5* and the TAV-overexpressing stable transgenic *Arabidopsis* line (*TAVox*) (Fig. 3). Note that, in *upf1-5*, the UPF1 mRNA level is severely reduced^61^. Three mRNAs were selected as potential NMD targets due to the presence of a long 3′-UTR and a premature termination codon on mRNAs: At1G01060, rps6 and smg7^7,20,62^. Three additional mRNAs were examined based on their predicted substrate potential for uORF-based NMD: At5G35490, At5G64430 and AtG36730^19^. All six mRNAs (Fig. 3a,b), including two additional mRNAs for AtG1390 and AtG22570 carrying unknown NMD-cis-elements^19^ (Fig. 3c), accumulated at higher levels in upf1-5 background and seedlings transgenic for TAV. We cannot exclude that TAV, as an RNA silencing suppressor, can usurp other molecular mechanisms to promote elevated expression levels of AtG1390 and AtG22570. Note that TAV may not only rescue the transcripts from NMD, but promote their further accumulation in some cases (see mRNAs for At1G01060 and At5G35490). In contrast, although mRNAs carrying AU-rich instability elements (ARE) within their 3′ UTRs (At2g4000, At1g72450 and At2g41640)^63^ remained elevated in the absence of the UPF1 protein, their levels were not affected or reduced in TAV-transgenic plants (Fig. 3d). Thus, NMD-sensitive transcripts that are stabilized in the seedlings depleted of NMD factors^19,64^ can specifically accumulate to high levels in TAV transgenic plants.

### TAV interferes with decay of the GFP-based NMD-sensitive reporter in *N*. *benthamiana*

**Figure 4:**
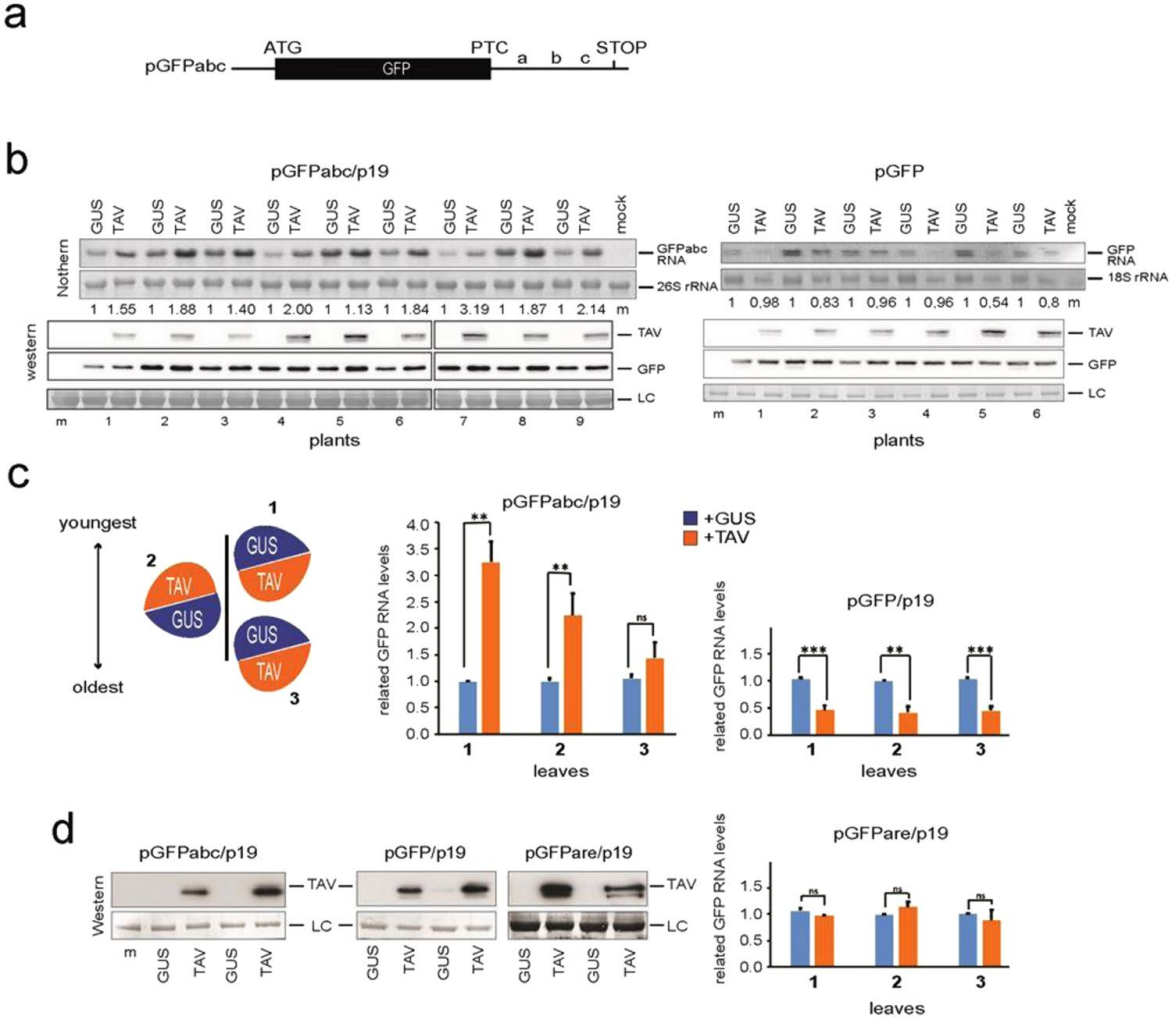
TAV stabilizes the NMD-sensitive target mRNA that contains the premature termination codon (PTC). (**a**) Schematic representation of GFPabc NMD-sensitive reporter (pGFPabc) containing PTC. (**b**,**c**) Agroinfiltration-based transient NMD suppression assay. (**b**) pGFPabc or pGFP (GFP-based reporter lacking signals for degradation) together with p19 were coinfiltrated into nine or six *N*. *benthamiana* plants, respectively, with one leaf each, along with pTAV (left half of leaf) or pGUS (right half of leaf). Samples were collected 4 days after infiltration (4 dpi). Total RNA was purified from each half of the leaf and subjected to Northern blot. Total protein extracts from the same leaf patches were analysed by western blotting to estimate TAV levels (two solid lines indicate that pGFPabc/p19-related samples were run on two gels under the same conditions). Coomassie brilliant blue staining of proteins and methylene blue staining of rRNA serve as a loading control for western blot and northern blot, respectively. Using ImageJ program the intensity of signal on northern blot was quantified and normalized to the rRNA loading control. The values of *GFPabc* RNA (left panel) and *GFP* RNA (right panel) in the presence of TAV were normalized against the mean value corresponding to GUS overexpression, which was set to 1.00, are presented at the bottom of northern blots. (**c**) Analysis of relative accumulation of *GFP* mRNAs by qRT-PCR in the infiltrated leaves at 4 dpi. Here, pGFPabc, or pGFP, or pGFPare (AU-rich degradation sensitive reporter) were infiltrated into three *N*. *benthamiana* plant with three leaves each (1, 2 and 3 as indicated on the left, where 1 is the youngest), together with p19, along with pTAV (left half of leaf) or pGUS (right half of leaf). *Nb ACT-b* (GI:380505031) and *Nb cdc2* (GI:849067) were used as reference genes. Values are expressed in arbitrary units and represent the mean +/− SD (n = 3). Each reporter statistical analysis was conducted independently for different categories of leaves; asterisks indicate a significant difference (*P < 0.05; **P < 0,01; ***P < 0,001, Student’s t test). (**d**) Western analysis of TAV was performed in duplicate by pulling plant material from three leaves for pGFPabc, pGFP and pGFPare. Loading controls (LC, RuBisCo) and mock (m) plants are shown.

We next sought to confirm whether TAV can indeed stabilize NMD-target mRNAs. In plants, the most-studied NMD-elicited mRNAs contain PTC as a result of aberrant splicing or mutations, or a stop codon placed in an environment unfavourable to translation termination^65^. It was shown that the mutant bean phytohemagglutinin (PHA) mRNA (PHA-m) is a critical NMD target due to a cis-NMD element located between the PTC of PHA-m and its original stop codon (referred to as the abc region, Fig. 4a)^66^. To directly test the effect of TAV on PTC-elicited NMD, we used a GFP reporter construct (pGFPabc) containing the abc region downstream of the GFP coding region stop codon that is transcribed to produce a strong and specific NMD target mRNA^66^. This reporter construct was coagroinfiltrated into *N*. *benthamiana* leaves with the plasmid construct expressing NMD suppressor candidate, TAV, on the leaf right half or the construct expressing β-glucuronidase (GUS; pGUS) on the left half of the leaf (Fig. 4b,c). As a control, the same experiment was performed with GFP expressing construct (pGFP) without any degradation signals. The p19 silencing suppressor from Tomato bushy stunt virus (TBSV), which inhibits silencing but does not interfere with NMD^66,67^ was co-infiltrated with each construct throughout our experiments to prevent the impact of RNA silencing on reporter mRNA accumulation. As shown in Fig. 4c, TAV overexpression leads to a higher steady state levels of *GFPabc* mRNA than co-expression of pGFPabc with GUS as manifested by both northern blot hybridization (Fig. 4b, left panels) and qRT-PCR analysis (Fig. 4c, upper panels). Importantly, the youngest leaves accumulated the highest levels of pGFPabc in the presence of TAV as compared to older mature leaves. By contrast, *GFP* RNA levels were somewhat lowered by TAV overexpression as compared with that of GUS (Fig. 4b,c, right panels). Notably, TAV overexpressed in plants appeared as multiple aggregates^68^ that may induce stress, and thus have a surprisingly negative effect on *GFP* mRNA accumulation. To test whether TAV exerts a general effect on mRNA decay, we introduced a 3′ UTR carrying an ARE downstream of the GFP ORF stop codon (pGFPare) and studied the accumulation of the *GFPare* mRNA with either TAV or GUS in agroinfiltration assay (Fig. 4d). No detectable changes in ARE-decay target mRNA levels were detected between TAV- or GUS-overexpressing leaf patches, suggesting that TAV, which is indifferent to stabilization of ARE-target signals, specifically stabilizes PTC-containing mRNAs.

**Figure 5:**
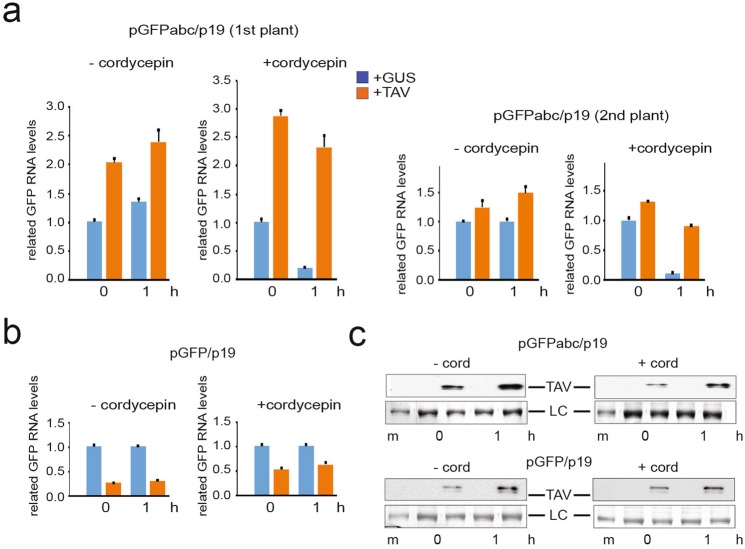
TAV stabilizes the NMD target mRNA in a transcription-independent manner. (**a**,**b**) Analysis of *GFPabc* and control *GFP* mRNA decay with either GUS or TAV using the agroinfiltration-based transient NMD supression assay in the absence and presence of cordycepin. pGFPabc, p19 or pGFP, p19 were coinfiltrated into *N*. *benthamiana* plants with one leaf each, along with pTAV (left half of leaf) or pGUS (right half of leaf). Leaves were infiltrated with buffer containing or not cordycepin at 4 dpi. Immediately after, leaves were detached and further incubated with or without cordycepin, respectively, for one hour. Relative accumulation of GFP mRNA in each leaf patch infiltrated with either pGFPabc (**a**) or pGFP (**b**) collected at the beginning and after one hour of incubation was anayzed by qRT-PCR; *Nb ACT-b* and *Nb cdc2* serve as internal standard. Data are presented as mean +/− SEM of three technical replicates. (**c**) TAV accumulation was demonstrated by western blot. Loading control (LC) is shown.

To rule out the possibility that NMD target stabilization was due to TAV-derived effects on transcription, we compared the impact of TAV or GUS on *GFPabc* and *GFP* transcript levels in plant patches infiltrated with or without cordycepin (Fig. 5). Cordycepin inhibits host transcriptional elongation when used in high concentrations^69^. Here, cordycepin nearly abolished auxin-induced gene expression in Arabidopsis seedlings (data not shown), in accordance with previous reports^70^. Figure 5a shows that steady state levels of *GFPabc* mRNA were drastically elevated in TAV-infiltrated patches but diminished in GUS-infiltrated patches in plants in which *de novo* transcription was inhibited. Indeed, a reduction in pGFPabc mRNA levels in plants containing GUS in the presence of cordycepin strongly suggests that cordycepin is functional and inactivates transcription. We concluded that TAV specifically inhibits NMD without rescuing transcription. In contrast, both TAV and GUS had a negligible effect on *GFP* mRNA levels lacking PTC. High TAV accumulation in *N*. *benthamiana* was detected throughout our experiments. In summary, our data show that TAV inhibits PTC-elicited mRNA degradation, but does not have an impact on RNA synthesis.

### TAV binds VCS to block mRNA degradation in response to the NMD signal

**Figure 6:**
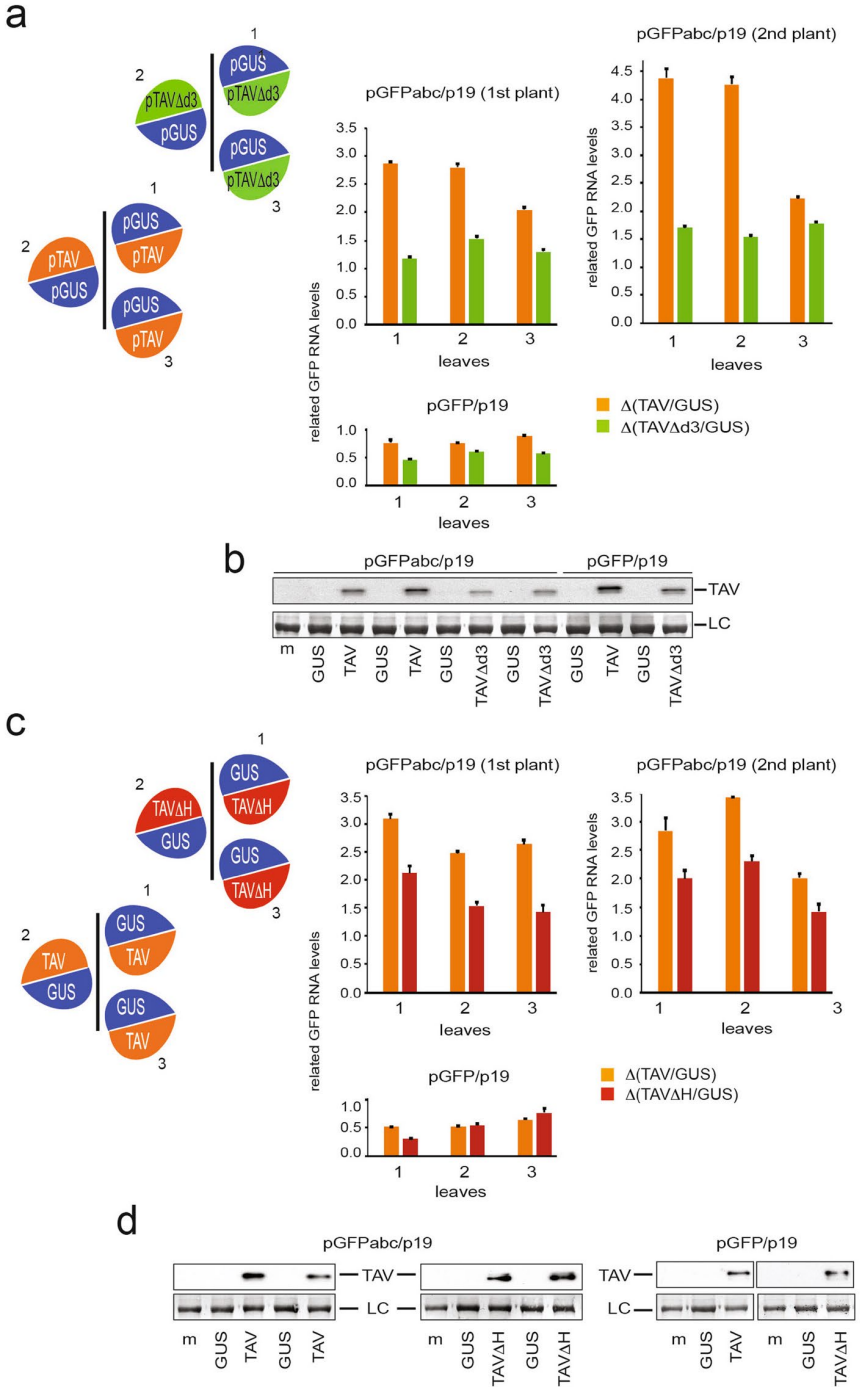
The d3 domain of TAV is responsible for stabilization of the PTC containing mRNA. Analyses of relative accumulation of *GFPabc* and *GFP* mRNAs by qRT-PCR in the leaves infiltrated with entire TAV or TAV deficient in VCS binding—TAV∆d3 and TAV∆H (see Fig. 1). Leaf patches were agroinfiltrated with pGFPabc or pGFP both with p19 and one of the following plasmids (**a**) pGUS, pTAV and pTAVΔd3 and (**c**) pGUS, pTAV and pTAVΔH as indicated on the left panel. RNA was extracted from the infiltrated patches at 4 dpi. qRT-PCR mean values of *GFPabc* RNA or *GFP* RNA accumulation in the presence of either (**a**) TAV or TAVΔd3 and (**b**) TAV or TAVΔH were normalized against the mean value for pGUS-infiltrated patches—(**a**) Δ(TAV/GUS), Δ(TAVΔd3/GUS) and (**b**) Δ(TAV/GUS), Δ(TAVΔH/GUS), respectively). *Nb ACT-b* and *Nb cdc2* serve as internal standard. Data are presented as mean +/− SEM of three technical replicates for each of three biological replicates (leaves 1–3) (**a**,**c**). Two plants are shown for pGFPabc/p19. Western analysis of TAV was performed in duplicate by pulling plant material from three leaves for pGFPabc and pGFP experimental set up with either TAVΔd3 (**b**) or TAVΔH (**d)**, pGFP/p19 – related samples were run on the same gel and two solid lines indicate removal of extraneous lanes; the original gel is available online as Supplemental Info). Loading control (LC) is shown.

NMD has been shown to activate subsequent decapping in yeast and human cells^71–74^, while the function of the decapping-dependent exonucleolytic pathway at the late step of NMD in plants is not well defined. Here we show that the PTC-elicited mRNAs accumulated to increased levels in TAV co-infiltrated leaves and associates with the decapping complex scaffold protein VCS, which suggest that TAV may downregulate the decapping machinery. Thus, we aimed to assess whether the TAV/VCS interaction contributes to TAV NMD inhibition. If this were the case, TAV lacking the d3 motif would fail to enhance stabilization of NMD-elicited mRNAs as the TAV d3 domain is responsible for VCS recognition (Fig. 1e). At 4 dpi, qRT-PCR analyses confirmed the reduced level of *GFPabc* mRNA stabilization by TAVΔd3 as compared with entire TAV, especially in the youngest leaves (Fig. 6a).

**Figure 7:**
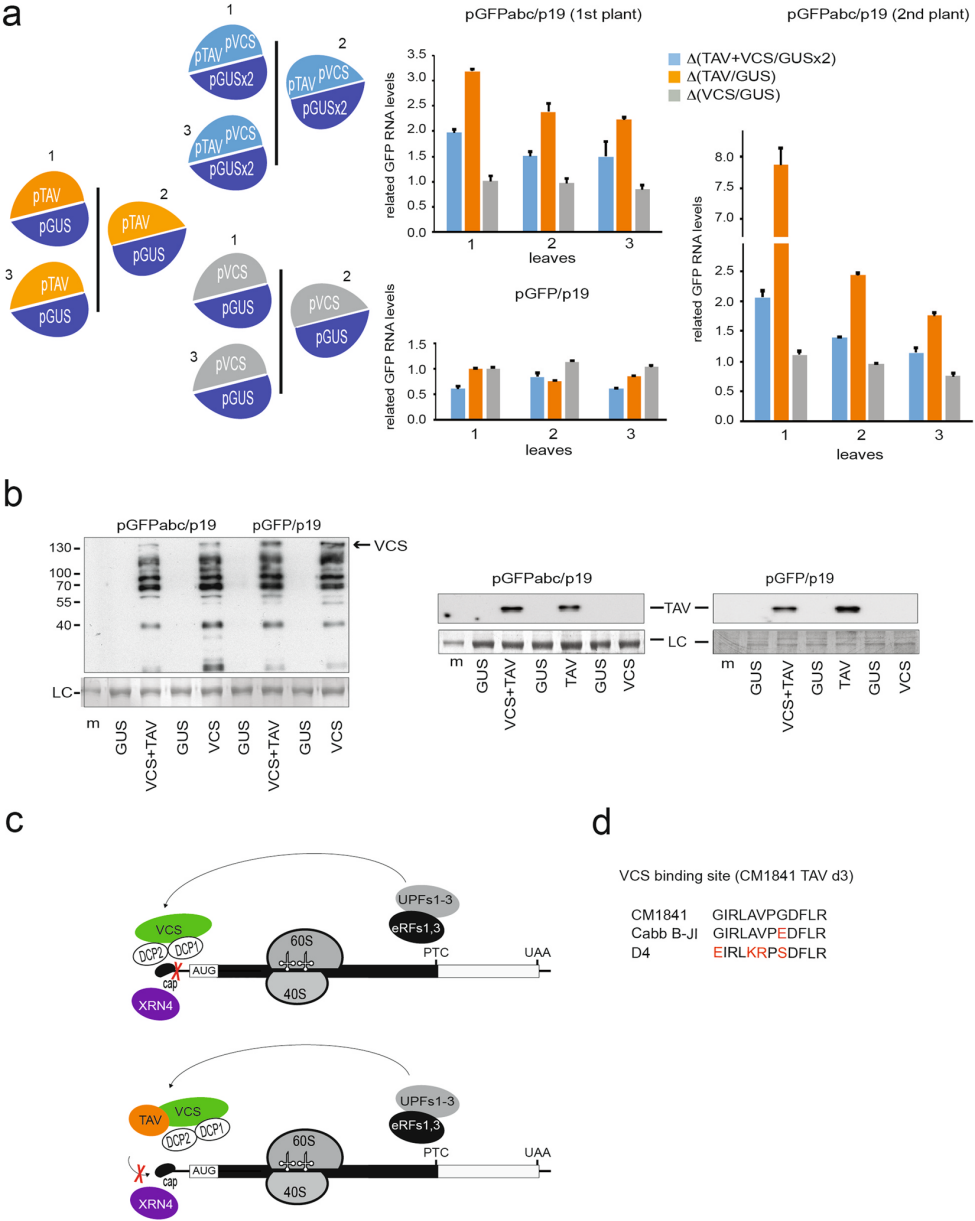
VCS overexpression suppresses stabilization of the PTC containing mRNA induced by TAV. (**a**) Analyses of relative accumulation of *GFPabc* and *GFP* mRNAs in conditions of TAV and VCS overexpression by qRT-PCR in the infiltrated leaves. pGFPabc/p19 or pGFP/p19 were co-infiltrated with either pTAV (left half of leaf) and pGUS (right half of leaf), or pVCS (left half of leaf) and pGUS (right half of leaf), or pTAV and pVCS (left half of leaf) and equal amounts of pGUS on the right half of leaf. qRT-PCR mean values of *GFPabc* RNA or *GFP* RNA accumulation in the presence of either TAV or VCS or both TAV and VCS were normalized against the mean value for corresponding pGUS-infiltrated patches. *Nb ACT-b* and *Nb cdc2* serve as internal standard. Data are presented as mean +/− SEM of three technical replicates for each of three biological replicates (leaves 1–3). Two plants are shown for pGFPabc/p19. (**b**) Western analysis of TAV and VCS accumulation in infiltrated patches are presented for pGFPabc and pGFP in each experimental set up. Loading control (LC) is shown. (**c**) Proposed model of NMD suppression by TAV. We suggest that the presence of PTC that triggers NMD leads to activation of the decapping machinery followed by a 5′–3′ exonuclease degradation. In the presence of TAV, the decapping step is blocked by TAV binding to VCS. DCP1, DCP2, VCS, TAV, 40S and 60S are indicated. (**d**) Comparison between 12 amino acid sequence motifs within the d3 domain found in TAV protein from CaMV strains CM1841, Cabb B-JI and D4.

Since the interaction between TAV and VCS is highly specific in the Y2H system, we reasoned that suppression of the decapping machinery would be sensitive to three amino acid substitutions within the VCS binding site of TAV. Indeed, alanine substitution of only three amino acids (PGD) within the D3 domain (TAVΔH) reduced the effect of TAV on GFPabc RNA stabilization as expected (Fig. 6b), while entire TAV, TAVΔd3 or TAVΔH deletion mutants were dispensable for accumulation of GFP mRNA lacking the PTC signal (Fig. 6a,b). Consistently, both mutants failed to interact with VCS in the Y2H system (Fig. 1f). All VCS, TAV and TAV mutants were well expressed in *N*. *benthamiana*, as verified by immunoblotting (Fig. 6a–c, bottom panels). Note that TAV overexpression did not significantly alter levels of transiently expressed VCS protein (Fig. 7b). Taken together, our data indicate a functional importance of the TAV/VCS interaction for the suppression of PTC.

We next studied whether expression of entire VCS protein in *N*. *benthamiana* would compete with endogenous VCS within the decapping complex for TAV binding. Strikingly, co-infiltration of pGFPabc with pVCS and pTAV led to three-fold suppression of the TAV effect on NMD (Fig. 7a). No effect on NMD suppression was obtained when pVCS alone was coinfiltrated with pGFPabc. This indicates that VCS alone may be competing with the endogenous complete decapping complex for TAV binding. Expression of VCS, VCS/TAV or TAV did not increase GFP mRNA levels with respect to pGUS (Fig. 7a, right panel). We propose that the decapping complex activity is suppressed in conditions of TAV overexpression due to sequestration of VCS by TAV. Our results suggest that the NMD pathway can be interrupted under specific conditions if the decapping pathway is blocked.

## Discussion

The work presented here suggests that the viral pathogenicity and host-range factor CaMV TAV contributes to suppression of cellular mRNA decay events, particularly the NMD process. We have identified the mRNA decapping activator VARICOSE as a novel TAV interacting partner. VCS binds the d3 domain of TAV CM1841, which is critical for TAV avirulence function. Our data suggest that CaMV TAV binding to VCS attenuates PTC-containing vector RNA degradation, thus suggesting tight coupling between the PTC-related NMD pathway and the decapping machinery in plants (Figs 4–7). Accordingly, TAV-overexpressing plants accumulate endogenous NMD target mRNAs with either uORFs within their leader regions, long 3′ UTRs and/or PTCs (Fig. 3). Since TAV activates target of rapamycin (TOR) protein kinase that specifically up-regulates translation of mRNAs that contain uORFs within their leader regions^50,51^, TAV could prevent activation of the uORF-elicited NMD pathway by improving efficiency of reinitiation events. However, TAV is inert for the cap-dependent translation initiation event^75^ and thus for a pioneer round of uORF-less mRNA translation that precedes function of the surveillance system. This study highlights another post-transcriptional control mechanism mediated by TAV, i.e., that of suppression of PTC-elicited RNA degradation, acting at the decapping step of the 5′ to 3′ exonuclease degradation pathway.

TAV controls reinitiation after long ORF translation^49,76^ via two distinguishable domains (MAV and MBD, Fig. 1c)^46,48,77^. The core transactivation domain MAV is responsible for interaction with TOR and reinitiation promoting protein RISP, while eIF3g and the 60S ribosomal protein L24 compete for the same binding site within the MBD domain^46,47,78^. The TAV domain d3, which is located toward the N-terminus of MAV, has been identified as the binding site for VCS, suggesting that the d3 domain as well as MAV and MBD are involved in posttranscriptional control. TAV binds the C-terminal HEAT repeat domain of VCS, which is implicated in VCS dimerization and binding of the decapping enzyme DCP2^79^. At this stage, we cannot conclude whether TAV binding to VCS interferes with DCP2 binding or decapping complex formation. Both TAV-dependent stabilization of PTC-containing mRNA (Fig. 4) and VCS binding to TAV (Fig. 1) are sensitive to a three amino acid change within the TAV d3 motif, suggesting specific function of TAV in the NMD pathway. We propose a preliminary model (Fig. 7c) where TAV interaction with VCS would prevent either DCP2 binding and/or VCS dimerization to interfere with the decapping machinery. This hypothesis supported by the fact that overexpression of VCS together with TAV in *N*. *benthamiana* leaves suppresses TAV-dependent stabilization of NMD-target mRNAs, presumably due to competition with endogenous decapping complex-bound VCS. However, given that VCS is located within PBs, cytoplasmic ribonucleoprotein foci implicated in mRNA storage, where TAV accumulates (Fig. 2), we cannot exclude that TAV can suppress the NMD-related pathway simply by depleting the decapping machinery stored in P-bodies. Strikingly, the VCS interaction network has been extended to include the recently reported complex formation between VCS and the stress-related ATP-dependent kinase SnRK2, which binds and phosphorylates VCS^80^. Recently, it was shown that turnip mosaic virus (TuMV) infection activates RNA decay to down-regulate TuMV RNA accumulation^81^. This antiviral activity is compromised by HC-Pro—viral suppressor of RNA silencing—that interacts and blocks XRN4 function, and VPg (the genome-linked viral protein) at the 5′end of TuMV RNA that can associate and thus sequester DCP2.

In yeast and human cells, NMD activates subsequent decapping^71–74^. Whether the NMD and decapping machineries are physically and functionally coupled in plants remains to be clarified. We observed earlier that TAV is detectable in 40S containing preinitiation complexes formed at the mRNA 5′ cap structure^47^, where it might interact with the decapping machinery. It was reported recently that NMD can act on aberrant mRNAs irrespective of whether cap is bound by the nuclear cap-binding complex (CBC) or eIF4E or an IRES in mammals^82,83^. Likewise, plants might not require CBC^84^ and mRNAs can undergo 5′–3′ decay while associated with ribosomes that undergo repeated translation rounds^85,86^, suggesting that TAV might interfere with the function of the decapping complex during steady state rounds of translation. Similarly, XRN4 accumulates in polysomes during XRN4-mediated degradation in response to heat-induced stress^87^. Interestingly, TAV aggregates colocalise with polysomes in the cytoplasm of *N*. *benthamiana* and *Arabidopsis* cells upon CaMV infection or transient expression^42,46,60^. Translation factor TAV is dispensable for stabilization of ARE-containing mRNAs, which might indicate that TAV function in NMD depends on the translation step and/or that the decapping step is dispensable for the ARE-mediated degradation pathway.

Studies of alterations in host gene expression induced by CaMV infection and TAV transgenic expression led to identification of several mRNA species up-regulated in CaMV-infected and/or TAV transgenic plants^88^. According to our analysis of available cDNA clones, at least three up-regulated mRNAs are loaded with one uORF and characterized by the long 3′UTR—features that can induce NMD decay. Of the transcripts that were unambiguously up-regulated in CaMV-infected and/or transgenic plants, the ATPase subunit A (AT1G78900)-encoding mRNA harbours a 34 codon uORF within the 5′UTR and 503-nt 3′UTR (data not shown). Here, TAV can play a dual function of promoting reinitiation after uORF translation via activation of TOR and supressing NMD via blocking function of the decapping complex.

The 35S pgRNA undergoes alternative splicing within 5′ and 3′ UTRs and ORFI-II regions^44,45^ and induces increased production of small RNAs in order to suppress the silencing machinery, implying that protection of the full-length 35S pgRNA from additional degradation by the cellular mRNA surveillance machinery is of crucial importance. The TAV Avr domain (aa 41–110) contributes to CaMV pathogenicity and host range^56,58,89,90^. Although the d3 motif is not essential for basic CaMV replication, it is a determinant of virulence, since deletion of this domain reduced infectivity of CaMV strain CM1841 and disease symptom development in turnip plants^56^. CaMV CM1841 virus lacking the d3 motif was shown to accumulate in systemic leaves of *A*. *thaliana* without developing disease symptoms^91^. It was suggested that the d3 domain contributes to silencing suppression^91^, and/or development of plant immune responses triggered, for example, by NB-LRR genes upon recognition of viral avirulence (Avr) proteins. Our previous studies confirmed neither importance of the d3 domain for TAV (P6) antisilencing activity, nor TAV-mediated suppression of plant immunity^36^. Instead, the TAV dsR domain previously shown to interact with the target of rapamycin (TOR)^48^ seems to induce suppression of SA-dependent autophagy^36^. Here, we discovered that, when CaMV TAV hijacks decapping components to downregulate NMD, the d3 motif can attenuate virus infectivity and symptom strength due to its effect on RNA stabilization. Accordingly, the 12-amino-acid motif is conserved between highly virulent strains (CM1841 and Cabb B-JI), while the TAV d3 motif from CaMV strain D4 is divergent (Fig. 7c) and likely fails to interact with VCS. This is supported by the fact that CaMV D4 displays only very mild symptoms in Arabidopsis^92^.

As general mechanisms of RNA turnover, cellular RNA quality control systems and RNA silencing function as major host defense mechanisms against viruses in plants, fungi and invertebrates^7,16–18,20^. We propose that CaMV can attack or destabilize key components of the cellular mRNA NMD machinery and this may provide mechanisms allowing high levels of viral RNA to be sustained in the cell. These data unveil a missing direct physical link between the NMD and mRNA decapping machineries and reveal that TAV employs an additional mechanism to maintain CaMV genome integrity. It will be interesting to explore if other infectious CaMV isolates, and perhaps other caulimoviruses, can interact with the cellular RNA degradation machinery.

## Materials and Methods

Plant material and growth conditions. The *Arabidopsis thaliana* lines *upf1-5* (SALK_112922) and TAV transgenic P6-CM1841 used in this study have been described previously^61,92^. *Nicotiana benthamiana* and *A*. *thaliana* plants were maintained in a greenhouse under conditions of 22 °C, 16 h light and 60% humidity.

### Plasmid construction

Yeast expression constructs were prepared using the pGAD-T7 and pGBK T7 vectors (Clontech). pGAD-TAV, pGBK-TAV, pGBK-NTAV and pGBK-CTAV have been described previously^47^. To create pGAD-VCS, the VCS coding sequence (AT3G13300) was amplified from an *A*. *thaliana* cDNA library with primers introducing *Eco*RI and *Sac*I restriction sites followed by insertion of the resulting fragment into the pGAD vector. To construct VCS domain-containing pGAD vectors, the appropriate portions of the VCS coding sequence were amplified with primers introducing *Nde*I/*Bam*HI or *Eco*RI/*Bam*HI restriction sites using pGAD-VCS as a template. pGBK vectors containing TAV deletion mutants derived from the pAATAVd1-d12 plasmids were kindly provided by Dr. K. Kobayashi^56^. The pGBK-TAV vectors containing three amino acid substitution mutations within the TAVd3 motif (A–J, Fig. 1f) were produced by site-directed mutagenesis of pGBK TAV using primers D3a–D3j.

To obtain constructs for transient expression of C-terminal GFP fusions with DCP1 (AT1G08370), DCP2 (AT5G13570) and VCS, the corresponding coding regions were amplified from an *A*. *thaliana* cDNA library with gene-specific primers containing attB recombination sites. The amplified products were recombined by Gateway cloning (Invitrogen) into the pB7FWG2 vector^93^. The constructs for transient expression of the C-terminal RFP-TAV fusion protein, and of the myc-tagged TAV, VCS, GUS, TAVd3 and TAV∆H proteins, were made by Gateway recombination using the entry vectors pH7RWG2^93^ and pGWB15^94^, respectively.

The reporter construct for the transient NMD assay pBin-GFPabc was kindly provided by Dr. D. Silhavy (Agricultural Biotechnology Institute, Godollo, Hungary). Binary constructs for the expression of p19 and mGFP in plants were kindly provided by Dr. P. Dunoyer (Institut de Biologie Moleculaires des Plantes, Strasbourg, France). The reporter construct containing an AU-rich signal within the 3′-UTR was obtained by cloning of the annealed primers carrying 11 ATTTA repeats, and *NheI*/*XbaI* restriction sites sticky ends; into *Nhe*I/*Xba*I digested pBin-GFPabc. All primer sequences are available online as Supplemental Info.

### Yeast two-hybrid analysis

Yeast two-hybrid (Y2H) analysis was performed as described in Thiébeauld *et al*.^46^. Yeast cells (strain AH109) were co-transformed by the small-scale lithium acetate yeast transformation method according to standard methods (Clontech). Transformants were selected on synthetic dropout minimal medium base containing 2% (w/v) glucose and dropout supplements lacking leucine (Leu), tryptophan (Trp), and adenine (Ade). The expression of BD and AD fusion proteins was confirmed by immunoblotting.

### Total RNA extraction and northern blotting

Equal amounts of deep-frozen leaf material were ground in a Precellys 24 homogenizer (Bertin Technologies). Total RNA was extracted using TRI-Reagent (Sigma-Aldrich) according to the manufacturer’s instructions and solubilized in sterile water. For northern blot analysis, 5 μg of total RNA was separated by denaturing gel electrophoresis, transferred to a nylon membrane (Hybond-N; Amersham Biosciences AB), cross-linked with UV light, prehybridized in PerfectHyb Plus buffer (Sigma-Aldrich) at 60 °C for 30 min and incubated overnight with the radioactive probe. The radioactive probe was generated using the Prime-a-Gene labeling system (Promega) with a PCR product corresponding to the 5′ 200 bases of GFP as template. After hybridization and washing, membranes were exposed to X-ray film for autoradiography.

### qRT-PCR

Total plant RNA was treated with DNase I (Promega) according to the manufacturer’s recommendations. 1 μg aliquots of total RNA samples were reverse-transcribed using SuperScript III reverse transcriptase (Invitrogen) with a mix of oligo(dT) primer and random hexamers. The cDNA was quantified using a LightCycler^®^ 480 SYBR Green I Master kit (Roche) and gene-specific primers. qRT-PCR was performed in 384-well optical reaction plates heated for 10 min at 95 °C, followed by 45 cycles of denaturation for 15 s at 95 °C, annealing for 20 s at 60 °C, and elongation for 40 s at 72 °C. A melting curve was performed at the end of the amplification in steps of 1 °C (from 95 °C to 50 °C). Transcript levels were normalized to that of *expansin-like A1* (*EXPLA1*, AT3G45970) and SAND family protein (*SAND*, At2g28390) for *A*. *thaliana*, and *Actin*, (*ACT-b* GI: 380505031) and cyclin-dependent kinase (CDC2, GI: 849067) for *N*. *benthamiana*. qRT-PCR primers sequences are presented in Supplementary Table 1.

### Protein analysis, SDS-PAGE, and immunoblotting

Leaf material (50 mg) was homogenized in 100 μl of 250 mM Tris pH 7.5 and 50 μl of 4 × Laemmli buffer, boiled for 5 min and centrifuged at 13200 rpm for 3 min. 5–15 µl of supernatant was resolved on 10% SDS-polyacrylamide gel electrophoresis, blotted to PVDF membrane (Immobilon-P, Merck Millipore) and subjected to immunoblot analysis. Anti-P6/TAV polyclonal antisera, previously obtained and tested in our laboratory, were used at a 1:20,000 dilution. Polyclonal antiGFP antibodies were kindly provided by Dr. D. Gilmer (IBMP, Strasbourg) and were used at a 1:12,000 dilution. Horseradish peroxidase-labelled secondary antibodies (Sigma-Aldrich) were used at a 1:20,000 dilution and visualised with Lumi-Light Plus ECL (Roche) and exposure to X-ray film.

### Transient NMD and AU-rich mediated inactivation assay

The transient NMD inactivation assay was performed as previously described with minor modifications^66^. The reporter vector and the RNA silencing suppressor p19 were co-expressed with or without indicated proteins by agroinfiltration in *N*. *benthamiana* leaves. All Agrobacterium strains were adjusted to a final optical density (OD600) of 0.2, while the strain delivering p19 was adjusted to an OD600 of 0.15. Samples for immunoblotting and northern blotting were collected at 4 dpi. Agroinfiltration was carried out as described in Voinnet *et al*.^67^.

### Confocal laser-scanning microscopy (CLSM)

Five-week old *N*. *benthamiana* plants were agroinfiltrated by agrobacterium strains adjusted to an OD600 of 0.2, and were used for confocal observations at 3 dpi. Confocal microscopy images were obtained with a Zeiss LSM700 inverted confocal laser microscope using a 40 × oil immersion objective. The excitation wavelengths for GFP and RFP detection were 488 and 561 nm, respectively.

### Transcriptional arrest

*N*. *benthamiana* leaves were agroinfiltrated as described for the NMD suppression assay. After 3 days, leaves were infiltrated with buffer containing 1 mM PIPES (pH 6.25), 1 mM sodium citrate, 1 mM KCl, 15 mM sucrose and 0.08% Silwet L-77 with or without cordycepin (150 mg/ml, Sigma)^70^. Immediately afterwards, leaves were detached and soaked in the equivalent buffer with or without cordycepin. For RNA and protein analysis, samples were collected at the time points 0 and 1 hour of incubation.

## Supplementary information

Supplementary data
