# Supplementary material for: Cauliflower mosaic virus transactivator protein (TAV) can suppress nonsense-mediated decay by targeting VARICOSE, a scaffold protein of the decapping complex
Source: Sci Rep. 2019 May 7;9:7042. doi: 10.1038/s41598-019-43414-0 (PMC6504953; doi:10.1038/s41598-019-43414-0)

## Supplementary information

# Cauliflower mosaic virus transactivator protein (TAV) can suppress nonsense-mediated decay by targeting VARICOSE, a scaffold protein of the decapping complex

Nina Lukhovitskaya and Lyubov A. Ryabova

## Table of contents

**1. Methods**

Supplementary Table 1

Supplementary Table 2

**2. Supplementary Figures**

Supplementary Figure S1

**3. Supplementary References**

**4. The unprocessed original scans for Figs 4-7**

## Methods

### Supplementary Table 1

| q-PCR primer | Target | Sequence 5'-3' | Reference |
|---|---|---|---|
| EXPL1F | At3G45970 | CTCCTCTGCCTCTGCTCTCTCT | |
| EXPL1R | At3G45970 | TCCGGCGAAGAAACTCGTA | |
| SANDF | At2g28390 | ATACTCGTCAACAGCAGAAA | |
| SANDR | At2g28390 | GTCACCCAACATAAAAGAAC | |
| RPS6-C-qF1 | rps6 | ATGTATTTTCAATGAGATCCCTTATC | (1) |
| RPS6-C-qR1 | rps6 | CCTTCTTAGTACCAGTGTTATGTTCAA | (1) |
| ARE1qFW | At1G72450 | TTCATCGATTCTTTGCTAAACG | |
| ARE1qREV | At1G72450 | ATCGATGGAGCAACCATCTC | |
| ARE2qFW | At2G4000 | TATGGAGCCGACGTATTTCC | |
| ARE2qREV | At2G4000 | TGACTCCAAAACTCTCCCAGA | |
| ARE3qFW | At2G41640 | GGCTTCTCTACTCTCTCCCTCCT | |
| ARE3qREV | At2G41640 | GGCTTCTCTACTCTCTCCCTCCT | |
| At5g22570FW | At5g22570 | CGATTCGGAAAAGATCAGGA | |
| At5g22570REV | At5g22570 | CGATAAGGAGAAACCGTTCG | |
| At4g13900FW | At4g13900 | GAATCCCAGTCTATGTGGTGCTC | |
| At4g13900REV | At4g13900 | TCTTGTTCTTGCTTCGTTGCTTC | |
| At1G01060FW | At1G01060 | ACAGGATGATTACCGTTCGTTTC | (2) |
| At1G01060REV | At1G01060 | CAATGGCAGTTATACTTGGAGGAG | (2) |
| At1G36730FW | At1G36730 | AAGATGCCGAGGATGATGAC | (2) |
| At1G36730REV | At1G36730 | AAGTGTCATGAGCCCCATTC | (2) |
| AT5G35490FW | AT5G35490 | TCCTCTTCCGATTCATTTGG | (2) |
| AT5G35490REV | AT5G35490 | TGCGTGACCAAGAATAGAGC | (2) |
| AT5G64430FW | AT5G64430 | TCACTTGTGGGCACTGAGAG | (2) |
| AT5G64430REV | AT5G64430 | AGCTGTGCGGATCTTGTCTT | (2) |
| SMG7-qF1 | smg7 | GCAGAGGGGGATTCCAGGAGCA | (1) |
| SMG7-qR1 | smg7 | TTGCAAGCTGATGATGTGGGTTTCC | (1) |
| EGFP-fwd | EGFP | TATATCATGGCCGACAAGCA | |
| EGFP-rev | EGFP | GAACTCCAGCAGGACCATGT | |
| CDC2-fwd | Nb CDC-2 | GTGTAGTGTACAAGGCTCGTGA | |
| CDC2-rev | Nb CDC-2 | TAGCTGTGCTTGGTACTCCCTC | |
| ACT-fwd | Nb actin | AAATTACTGCACTTGCTCCTAGC | |
| ACT-rev | Nb actin | CAATCCAGACACTGTATTTCCTCTC | |

**Supplementary Table 2**

| | | |
|---|---|---|
| VCSEIFW | Cloning of VCS into YTH vectors | CAGAATTCGCGTCTTCTCCTGGTAATAC |
| VCSSacIREV | | CAGAGCTCCCTCATTTGCAACCCATAAGC |
| VCS1NdeFW | Cloning of VCS domains into YTH vectors | CACATATGGCGTCTTCTCCTGGTAATAC |
| VCS1EIREV | | CAGAATTCTAAGCACAAGTCTAATGTATAC |
| VCS2EIFW | | CAGAATTCTGCTTGCCACCACCTATAG |
| VCS2BamREV | | CAGGATCCTGTCATGGGATGACTTAAAC |
| VCS3EIFW | | CAGAATTCGATTTACTTCCT AGTTGTTAG |
| VCS3BamREV | | CAGGATCCATTTGCTCCAGCAGCTGCGAGAG |
| VCS4EIFW | | CAGAATTCTCTGGTGGGTCTAATTCCTTG |
| VCS4BamREV | | CAGGATCCTTTGCAACCCATAAGCATGG |
| D3AaFW | Cloning of TAV D3 three amino acid substitution mutants into YTH vectors | ACAAATCCGGCGGCGGGTAATATCTTGCCAAAAG |
| D3AaREV | | CTTTTGGCAAGATATTACCCGCCGCCGGATTTGT |
| D3BaFW | | CCGTTGATGGCTGCTGCCGCGCCAAAAGATATG |
| D3BaREV | | CATATCTTTTGGCGCGGCAGCAGCCATCAACG |
| D3CaFW | | GGCTAATATCTTGGCAGCAGCTATGAACCCAGTTCAAAC |
| D3CaREV | | GTTTGAACTGGGTTCATAGCTGCTGCCAAGATATTAGCC |
| D3DaFW | | CTTGCCAAAAGATGCGGCCGCAGTTCAAACTGGG |
| D3DaREV | | CCCAGTTTGAACTGCGGCCGCATCTTTTGGCAAG |
| D3EaFW | | GATATGAACCCAGCTGCAGCTGGGATAAGGCTTG |
| D3EaREV | | CAAGCCTTATCCCAGCTGCAGCTGGGTTCATATC |
| D3FaFW | | CCAGTTCAAACTGCGGCAGCGCTTGCAGTGCCAG |
| D3FaREV | | CTGGCACTGCAAGCGCTGCCGCAGTTTGAACTGG |
| D3GaFW | | CAAACTGGGATAAGGGCTGGAGCGCCAGAGGACTTTTTAC |
| D3GaREV | | GTAAAAAGTCCTCTGGCGCTCCAGCCCTTATCCCAGTTTG |
| D3HaFW | | GATAAGGCTTGCAGTGGCAGCGGCCTTTTTACGTCCTC |
| D3HaREV | | GAGGACGTAAAAAGGCCGCTGCCACTGCAAGCCTTATC |
| D3IaFW | | GTGCCAGAGGACGCTGCAGCTCCTCATCAGGG |
| D3IaREV | | CCCTGATGAGGAGCTGCAGCGTCCTCTGGCAC |
| D3JaFW | | GACTTTTTACGTGCTGCTGCGGGAATTCCA |
| D3JaREV | | TGGAATTCCCGCAGCAGCACGTAAAAAGTC |
| TAVattBfw | Cloning of TAV into gateway vectors | GGGGACAAGTTTGTACAAAAAAGCAGGCTTCATGGAGAACA TAGAAAAACTC |
| TAVattBrev | | GGGGACCACTTTGTACAAGAAAGCTGGGTCTCAATCCACTTG CTTTGAAGAC |
| TAVattBtyrrev | | GGGGACCACTTTGTACAAGAAAGCTGGGTCGTAATCCACTTG CTTTGAAGAC |
| attBVCSfw | Cloning of VCS into gateway vectors | GGGGACAAGTTTGTACAAAAAAGCAGGCTTCATGGCGTCTTC TCCTGGTAATAC |
| attBVCSrev | | GGGGACCACTTTGTACAAGAAAGCTGGGTCTCATTTGCAACC CATAAGCATG |
| attBVCStyrrev | | GGGGACCACTTTGTACAAGAAAGCTGGGTCGTATTTGCAACC CATAAGCATG |
| attBDCP1fw | Cloning of DCP1 into gateway vectors | GGGGACAAGTTTGTACAAAAAAGCAGGCTTC ATGTCTCAAA ACGGGAAGATAATC |
| attDCP1rev | | GGGGACCACTTTGTACAAGAAAGCTGGGTCTCATTGTTGAAG TGCATTTTG |
| attDCP1tyrrev | | GGGGACCACTTTGTACAAGAAAGCTGGGTCGTATTGTTGAAG TGCATTTTGTAAAG |
| attBDCP2fw | Cloning of DCP2 into gateway vectors | GGGGACAAGTTTGTACAAAAAAGCAGGCTTCATGTCGGGCCT CCATCGATCATC |
| attBDCP2rev | | GGGGACCACTTTGTACAAGAAAGCTGGGTCTCAAGCTGAATT ACCAGATTCC |
| attBDCP2tyrrev | | GGGGACCACTTTGTACAAGAAAGCTGGGTCGTAAGCTGAATT ACCAGATTCCAAC |
| AUUUA FW | Cloning of ARE degradation reporter | GCGGATCCGATCTAGTAATATTTAATTTATTTATTTATTTATTT ATTTATTTATTTATT TATTTAAAGGATCGCTAGCCG |
| AUUUA REV | | CGGCTAGCGATCCTTTAAATAAATAAATAAATAAATAAATAA ATAAATAAATAAATT AAATATTACTAGATCGGATCCGC |

## 2. Supplementary Figures

### Supplemental Figure S1

```
                     ***.+++.+.++.+++++*. ++*+.++*...*...    .+*...+.**.+.+.+.**.+
VCS   (      1) MASSPGNTNPHNTPPFDLGILFKPSSNPYPPPAASypppTGPFLHNQYDQQHYAPPGISA
VCR   (      1) MASSPGNTNPHNPPPFDLGTIFKPSSNPYPPP-------TGPFLNNQYNQQLYAPPGIAA
Ge-1  (      1) MASC-ASIDIEDATQ-HLRDILK-LDRPAGGPSAE-----SPRPSSAYNGDL---NGLLV

                .*+*+.. +.*.*+*+..*+*.+.**+.++.**+*.*+++.++++.+.++*.++  .+.
VCS   (     61) QPSPVTQQQQDVSSSSAATNLHPQRTLSYPTPPLNLQSPRSNHNPGTHILALLNNtnNGA
VCR   (     54) QPSPVNQTQQDVSSSSSATNLQPQRTLSYPTPPLNPQSPRVNHNPGTHILALLNN--GGA
Ge-1  (     50) -PDPL--CSGD-STSANKTGL---RTM----PPINLQEKQV--------ICLSGD-----

                 ..+..+.+      ++.....++++*..* *+. .++*..++*+.+*..*.+*..+++
VCS   (    121) pVANQEPSHqlpvvnHNEIARSFPGGSGPIRVPSCKLPKGRRLIGEHAVYDVDVRLQGEI
VCR   (    112) -VANQEPSH------HNEIARAFPGGSGPIHVPSGKMPKGRRLVGEHAVYDVDVRLQGEI
Ge-1  (     86) ------------------------DSSTCIGI----LAKEVEIV---ASSDSSISSKARG

                +.++++*.*****+.*++...*+.****+.++++++    **+. *******+*.*++++*
VCS   (    181) QPQLEVTPITKYGSDPQLVVGRQIAVNKVYICYGLK----GGN-IRVLNINTALRSLFRG
VCR   (    165) QPQLEVTPITKYGSDPQLVLGRQIAVNKVYICYGLK----GGS-IRVLNINTALRSLFRG
Ge-1  (    115) SNKVKIQPVAKYDWEQKYYYGNLIAVSNSFLAYAIRaannGSAmVRVISVSTSERTLLKG

           ++..*+***...+. .**.++..*++****+..+..+.....+****  .+.++*...*
VCS   (    236) HSQRVTDMAFFAEDVDMLASVSLDGKVFVWKISEGSEGEDQPQITGKI—-VLALQILGEE
VCR   (    220) HSQRVTDMAFFAEDVHLLASVSLDGKVFVWKISEGSEGDEQSQITGKI—-VVALQILGEE
Ge-1  (    175) FTGSVADLAFAHLNSPQLACLDEAGNLFVWRL---------ALVNGKIqeEILVHIRQPE
                         WD40 motif
                +*   ++  .***+         ++*.+++..+*+++.+.*+.*.++*...+++ *++++..+
VCS   (    294) DT--KH-PRVCW---------HCHKQEILVVSIGKHVLRIDTTKVGRGEVF-SAEAPLQC
VCR   (    278) DT--KH-PRVCW---------HCHKQEILVVSIGKHVLRIDTTKVGRGEVF-SAEAPLQC
Ge-1  (    226) GTplNHfRRIIWcpfipeeseDCCEESSPTVAL----LHEDRAEVWDLDMLrSSHSTWPV

                 ++++.+*..**..*+. ++* +++...*+.*+++*.**.****    *.+ +*   *...
VCS   (    341) PLDKLIDGVQIVGKHDGEVTDLSMCQWMTTRLVSSSVDGTIKIWQ---DRKAQP--LVVL
VCR   (    325) HLDKLIDGVQIVGKHDGEVTDLSMCQWMTTRLVSSSVDGTVKIWQ---DRKTQP--LVVL
Ge-1  (    282) DVSQIKQGFIVVKGHSTCLSEGALSPDGTV-LATASHDGYVKFWQiyiEGQDEPrcLHEW
                                 WD40 motif
                +**** *++...*..+     *+.*.+.+*****+.******.+.+..++..+.+..**
VCS   (    396) RPHDGHPVSSATFVTS-----PERPDHIILITGGPLNREMKIWVSAGEEGWLLPADAESW
VCR   (    380) RPHDGLPVNSAIFVTS-----PERPDHIILITGGPLNREIKIWVSAGEEGWLLPADTESW
Ge-1  (    341) KPHDGRPLSCLLFCDNhkkqdPDVPFWRFLITGADQNRELKMWCT------------VSW

                .*.***    **.****  *.+.+++++..+.**.*..*+*++++++++*.++*.+++++.
VCS   (    451) RCTQTL----DLKSSTE-PRAEEAFFNQVIALSEAGLLLLANAKRNALYAVHLDYGSSPV
VCR   (    435) RCTQTL----DLKSSTE-PQAEKAFFNQVIALSEAGLLLLANARRNAIYSVHLDYGSSPV
Ge-1  (    389) TCLQTIrfspDIFSSVSvPPSLKV----CLDLS-AEYLILSDVQRKVLYVMELLQNQEEG

                 + ++.+***.+*.*****                             .**+     * +..
VCS   (    506) GTRMDYLSEFTVTMPILSF---------------------------IGTN-----DPPEE
VCR   (    490) ETLMDYLSEFTVTMPILSF---------------------------IGTN-----DHPEE
Ge-1  (    444) HACFSSISEFLLTHPVLSFgiqvvsrcrlrhtevlpaeeendslgaDGTHgagamESAAG

           ..*******.*+*   *     ..*+. ++.*.* .+.+ *..+...**... .*+. ...
VCS   (    534) PIVKVYCVQTLAIQ--Q----YTLDLCLCLPPPIENMGLEKSDSSVSREANLVEGM-SEP
VCR   (    518) PFVKVYCVQTLAIQ--Q----YTLDLFLCMPPPRENVGFEKSDSTVSREANLVEST-LET
Ge-1  (    504) VLIKLFCVHTKALQdvQirfqPQLNPDVVAPLP-THTAHEDFTFGESRPELGSEGLgSAA

           .*.+*.**..* +++.*+..++ .**....*..*    ++ .*+.*.+++ ++++   +++
VCS   (    587) SGLKPTDLPSVDSVPKPSIIVN-RSESANKLSFP----SAEATSQAIVPP-NGEP--KTS
VCR   (    571) SGMKPTELPSVGSVPKPSILVN-RSENANMLSFPagpaSAGITPPAIVPP-NGEP--KTS
Ge-1  (    563) HGSQP-DLRRIVELPAPADFLSlSSETKPKLMTP----DAFMTPSASLQQiTASPssSSS
```

```
               *.+*.+*...*+          .*  *++   *    ***+*..+*+*..*..++ + .+
VCS   (   639) GLPSQTSGAGSA----------YATLPQL---P----LSPRLSSKLSGYHTPVEAIE-PV
VCR   (   627) GMPSETSDVDSA----------YAPSPQL---P----LSPRLSSKLSGYHTPVEAFE-QV
Ge-1  (   618) GSSSSSSSSSSSltavsamsstSAVDPSLtrpPeeltLSPKL--QLDGSLTMSSSGSlQA

               .*++ *.*..++ **.   +   *. +.++ .+**+++**.  ..+.+ +.*.++**.++
VCS   (   681) IPHHELGGKTPS-ADYSVDR---QM-DAVGERNLDVSSVEEISRSKDS-NVTPDDDVSGM
VCR   (   669) LPHHKLGGKTSS-ADYFYVR---QT-DDVGGRNLDVSSVEENCRSKDT-NVTPDDDVSGI
Ge-1  (   676) SPRGLLPGLLPApADKLTPKgpgQVpTATSALSLELQEVEPLGLPQASpSRTRSPDV--I

               .*+*+.++++.+.*++**.* .*. **+..++..++..+..+.+. ++..+* + *+...
VCS   (   735) RSPSAFFKHPTHLVTPSEIL-MGV-SSAEASITTEDRRDRDANIQDVNNDPR-DTEVEVK
VCR   (   723) RSPSAFFKQPTHLVTPSEIL-MGV-SSTEASITTEDKRDRDANIEEVNNDAR-GLEVELK
Ge-1  (   734) SSASTALSQDIPEIA-SEALsRGFgSSAPEGLEPDSMASAASALHLLSPRPRpGPELG-P

               +++.. ...+*+ .* +. .*. .++..+..+.**+...+.+++** ..+.+*    *.
VCS   (   792) EISEARSTQNGE-INDHDETENCTSENREKVFCSQVSNLSTEMARD-CYPSTE----GTF
VCR   (   780) EVGEAQTSQNGE-INYHETTENHTSESRENIFCSQASNLSTEMARD-RHPITE----GSA
Ge-1  (   792) QLGLDGGPGDGDrHNTPSLLEAALTQEASTP-DSQVWPTAPDITREtCSTLAEsprnGLQ

               .+..*.**.+ *   ++..*      *.+.*..+  *++   ..+ .. . . ...+*..
VCS   (   846) IPGESKAYGQ-P---IKAGD------ESGVDSRGGPAKL---------------LKGKKQ
VCR   (   834) IPGDSMAYGQ-P---LQAGD------ERGLDSRDVSAKL--PESGSSSGLVATNSKGKKQ
Ge-1  (   851) EKHKSLAFHRpPyhlLQQRDsqdasaEQS-DHDDEVASLasASGGFGTKVPAPRLPAKDW

                                                   → α-helix/coil C-terminus
               *+*+*  .+*.*   *+.+++.*.+++.++*..+++.+*..*..*.*+ ..*+.+.++..
VCS   (   881) KAKNS--QGPGL---SSTSSNVANLADSFNEQSQSLSHPMTDLLPQLL-AMQETMNQVMA
VCR   (   882) KAKNS--QGPGL---SSTSSNVANLADSFNEQSQSLNHPMADLLPQLL-ALQETMTQVMA
Ge-1  (   910) KTKGSprTSPKLkrkSKKDDGDAAMGSRLTE--HQVAEPPED-WPALIwQQQRELAELRH

               **+*+.++*.+...*+. .....*+.++...**++.+++.+..+.+ **+..+.*+**.*
VCS   (   935) SQKEMQRQLSNAATGPIGKESKRLEVALGRMIEKSSKSNADALWARIQEETVKNEKALRD
VCR   (   936) SQKEMQRQLSNAVTGPIVKEGKKLEVALGRMIEKSSKSNADALWAHFQEEAVKNEKALRD
Ge-1  (   967) SQEELLQRLCTQLEG--------LQSTVTGHVERALETR------HEQEQR-RLERALAE

               + *   *+.+..*+..*+.* +*+   +*++++.*. ..*.*.++*.+ *+..+..+*+.
VCS   (   995) HAQ---QIVNATTNFMSKEL-NAM---FEKTIKKEL-AAIGPALARSVVPVIEKTVSSAI
VCR   (   996) HGQ---QIMNETTNFTSKEL-NAM---FEKTMKKEF-ASVGPTLARVVTPVIEKTVSSAI
Ge-1  (  1012) GQQrggQLQEQLTQQLSQALsSAVagrLERSIRDEIkKTVPPCVSRSLEPMAGQLSNSVA

               *+ +..+*..++.*+++ **.* .*. +***...*..+*.++. +*.+.*++.+*.*+*+
VCS   (  1047) TE-SFQRGIGDKAVNQLDKSVNIKLEATVARQIQAQFQTSGKQALQEGLRSSVESSVIPS
VCR   (  1048) TE-SFQRGIGDKAVNQLEKSVNSKLETTVARQIQAQFQTSGRQVLQEGLRSSMESSVIPS
Ge-1  (  1072) TKlTAVEGSMKENISKLLKSKN--LTDAIAR---AAADTL-QGPMQAAYREAFQSVVLPA

               **++*++**+**++.++.*       + .*..+.+.* +++.+.+.+.*+..*++.+*++
VCS   (  1106) FEKACKAMFDQIDSAFQKG-------IAEHTNAAQQRFDSGHSQLAHTLKESITSASSVA
VCR   (  1107) FERSCKTMFEQVDSTLQKG-------IGKHTSATQQRIDSGQSQLAHTLRETISSASSVT
Ge-1  (  1126) FEKSCQAMFQQINDSFRLGtqeylqqLESHMKSRKAREQEAREPVLAQLRGLVSTLQSAT

               +.+...+*.+.*. +  ..  *   **  +.+++*++   .*++            ++++
VCS   (  1159) QALSRELAETQRNLLALAAAGAnsGGS--NSLVTQLS---GGPL------------GALL
VCR   (  1160) QALNRELAESQRNRLALTAA-----GS--NPLVTQLS---NGPL------------GALL
Ge-1  (  1186) EQMAATVAGSVRAEVQHQLHVA--VGSlqESILAQVQrivKGEVsvalkeqqaavtSSIM

               +.+         +*.+*   +.+++.+*+++..++++*.+**..+*+++*.*+*+.**..
VCS   (  1202) EKV---------EAPMD---PTTELSRLISERKYEESFTSALQRSDVSIVSWLCSQVDLR
VCR   (  1198) EKV---------EAPMD---PTTELSRLISERKYEESFTSALQRSDVSIVSWLCSQVDLR
Ge-1  (  1244) QAMrsaagtpvpSAHLDcqaQQAHILQLLQQGHLNQAFQQALTAADLNLVLYVCETVDPA
```

```
                .++++.*.****.*****+*****+*++..*+.**.*+.*+*..++.**.+...*..+*+.
VCS   (   1250) GLLAMNPLPLSQGVLLSLLQQLACDISKDTSRKLAWMTDVVAAINPSDQMIAVHARPIFE
VCR   (   1246) GLLAMNPLPLSQGVLLSLLQQLACDISTDTSRKLGWMTDVVTAINPSDQMIAVHARPIFE
Ge-1  (   1304) QVFGQPPCPLSQPVLLSLIQQLASDLGTRTDLKLSYLEEAVMHLDHSDPITRDHMGSVMA
                             Ψ(X2-3)
                **    **+*  ++*.*+.*+.+..+.*++*.++.+..++.
VCS   (   1310) QV----YQIL--HHHRNAPGSDVSAIRLIMHVINSMLMGCK
VCR   (   1306) QV----YQIL--HHHRNAPGSDVSAVRLIMHVINSLLMSCK
Ge-1  (   1364) QVrqklFQFLqaEPH-NSLGKAARRLSLMLHGL--VTPSLP
```

**Supplemental Figure 1. Arabidopsis (At) VCS and AtVCR aligned with their homolog in Homo sapiens (h), Hedls/Ge-1.**

WD40 and $\Psi(X_{2\text{-}3})$ motifs predicted for the VCS and VCR are shown in yellow and green, respectively. The start of the C-terminal region with alpha-helical fold is indicated by arrow. The degree of sequence similarity was estimated as 43.5%. The meaning of signs at the top of the alignment is following: ' ' - the average weight of column pair exchanges is less than weight matrix mean value; '.' - is less than mean value plus one SD; '+' - is less than mean value plus two SD; '*' - is more than mean value plus two SD.

## 3. Supplementary References

**References**

1. Gloggnitzer, J. *et al.* Nonsense-mediated mRNA decay modulates immune receptor levels to regulate plant antibacterial defense. *Cell Host Microbe* **16,** 376–390 (2014).
2. Garcia, D., Garcia, S. & Voinnet, O. Nonsense-mediated decay serves as a general viral restriction mechanism in plants. *Cell Host Microbe* **16,** 391–402 (2014).

# Fig. 4b

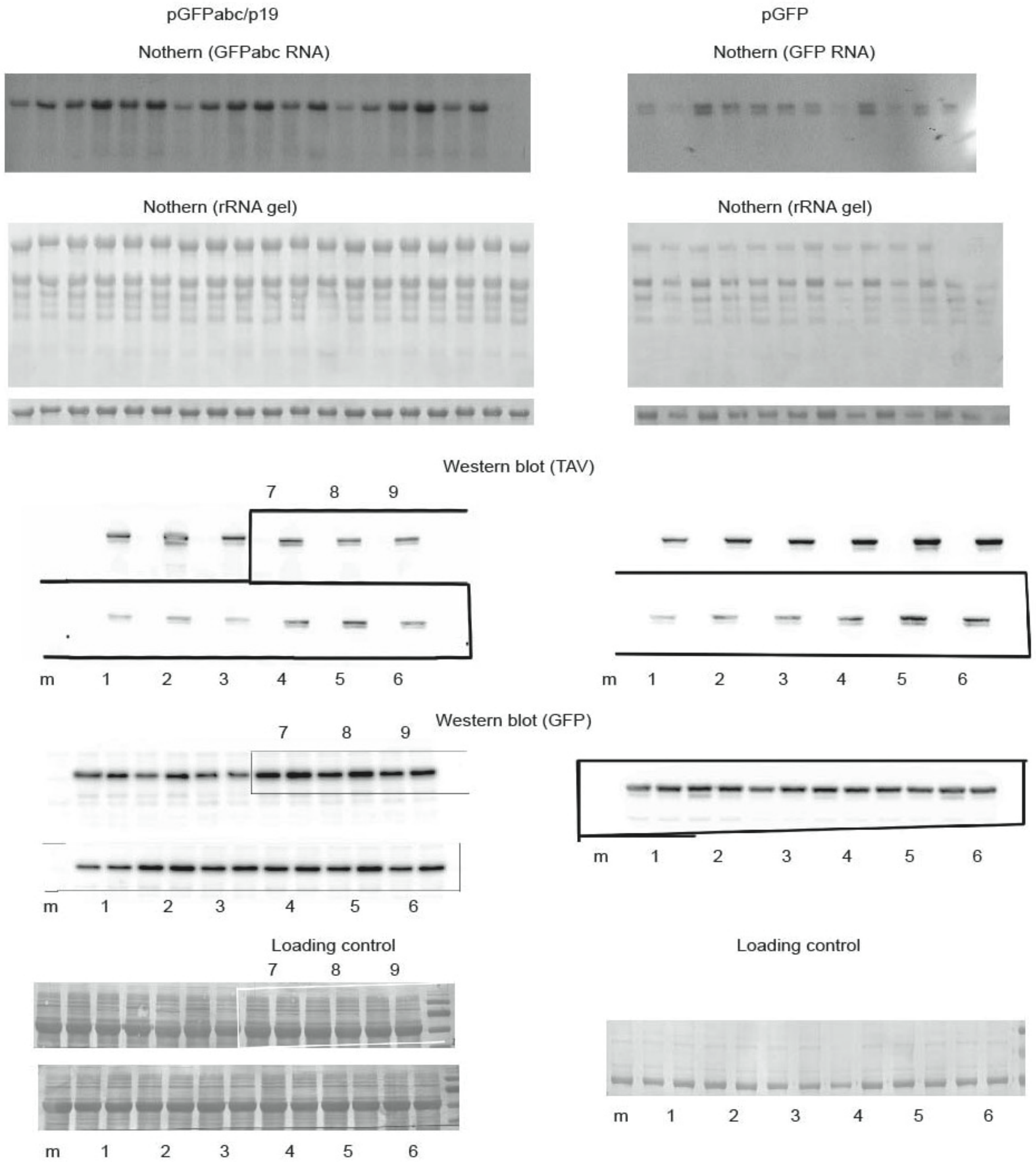
pGFPabc/p19
Nothern (GFPabc RNA)
Nothern (rRNA gel)
pGFP
Nothern (GFP RNA)
Nothern (rRNA gel)
Western blot (TAV)
7 8 9
m 1 2 3 4 5 6
m 1 2 3 4 5 6
Western blot (GFP)
7 8 9
m 1 2 3 4 5 6
m 1 2 3 4 5 6
Loading control
7 8 9
m 1 2 3 4 5 6
Loading control
m 1 2 3 4 5 6

# Fig. 4d

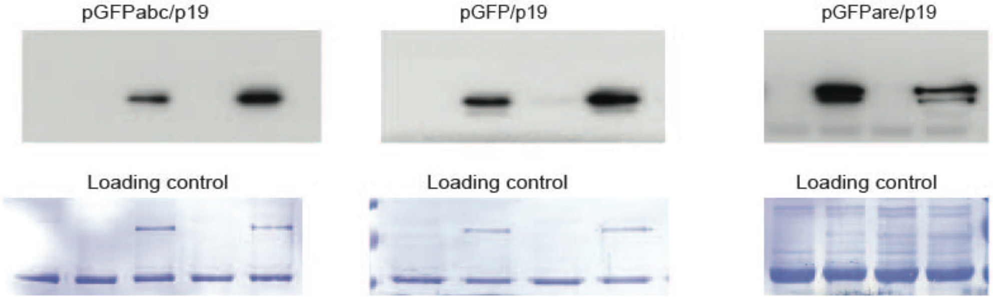
pGFPabc/p19
pGFP/p19
pGFPare/p19
Loading control
Loading control
Loading control

Fig. 5c

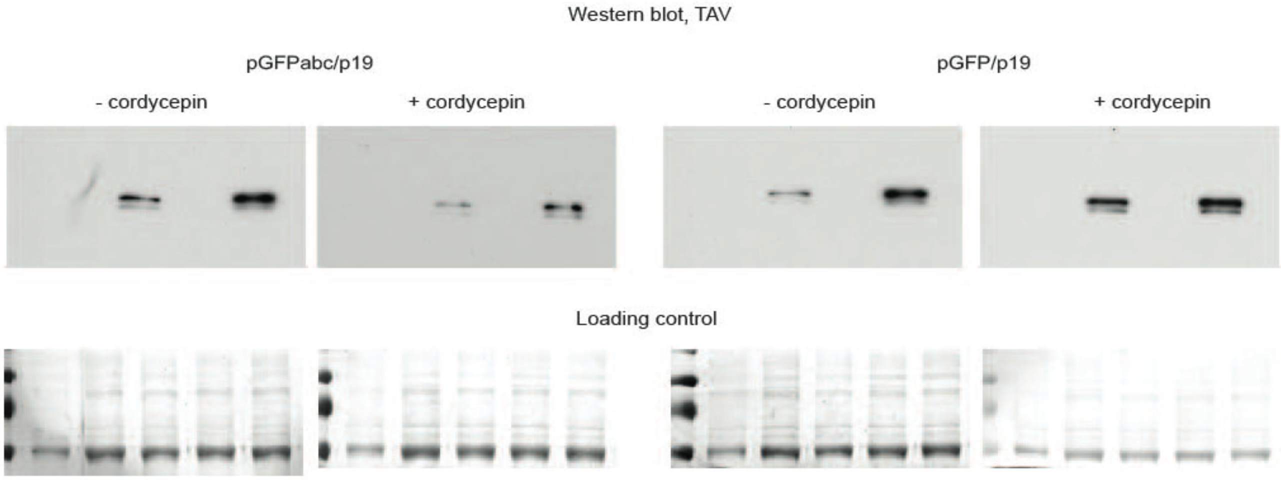
Western blot, TAV
pGFPabc/p19
pGFP/p19
- cordycepin
+ cordycepin
- cordycepin
+ cordycepin
Loading control

# Fig. 6b

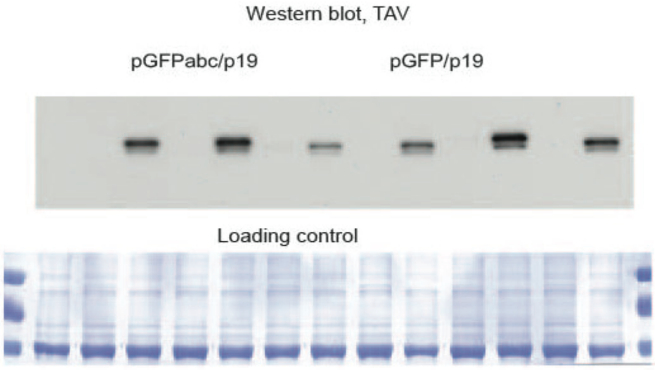


# Fig. 6d

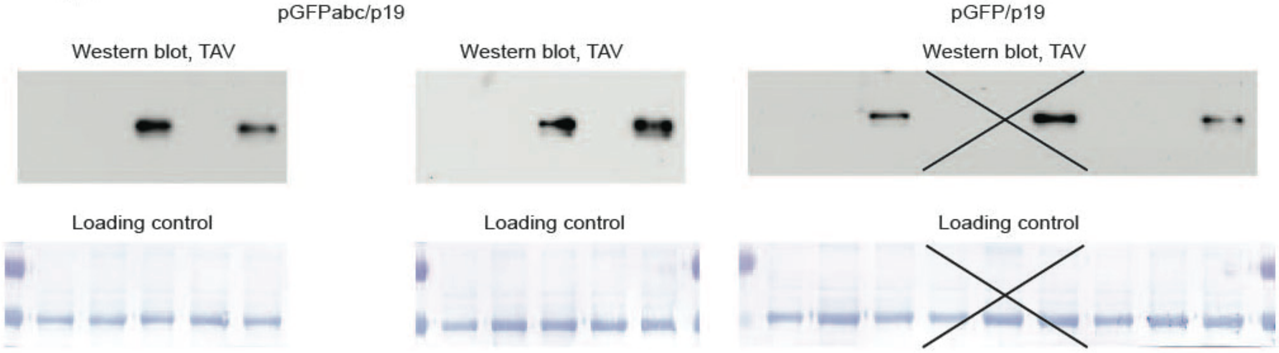


# Fig. 7b

Left bottom panel

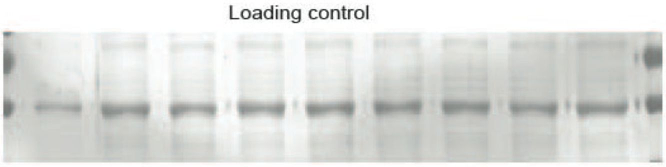


Right panels

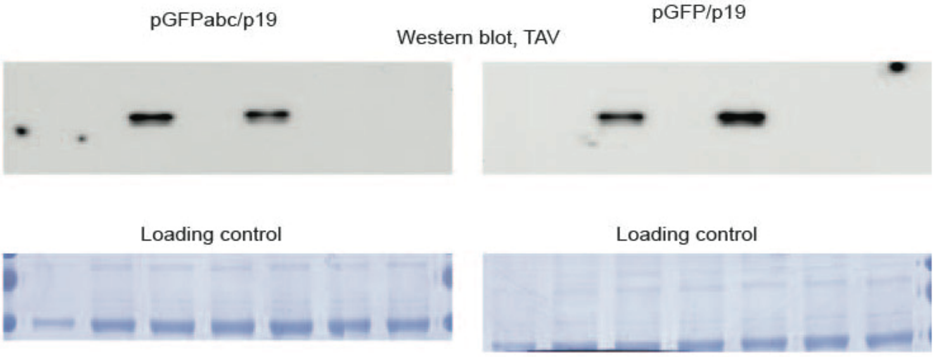

Supplement: Supplementary file 1 — Supplementary data [file 41598_2019_43414_MOESM1_ESM.pdf]
